# Discovery and evolution of novel hemerythrin genes in annelid worms

**DOI:** 10.1186/s12862-017-0933-z

**Published:** 2017-03-23

**Authors:** Elisa M. Costa-Paiva, Nathan V. Whelan, Damien S. Waits, Scott R. Santos, Carlos G. Schrago, Kenneth M. Halanych

**Affiliations:** 1 Departamento de Genética, Laboratório de Biologia Evolutiva Teórica e Aplicada, Universidade Federal do Rio de Janeiro, Rio de Janeiro, RJ Brazil; 2 Department of Biological Sciences, Molette Biology Laboratory for Environmental and Climate Change Studies, Auburn University, Auburn, AL 36849 USA; 3 Warm Springs Fish Technology Center, U.S. Fish and Wildlife Service, 5308 Spring ST, Warm Springs, GA 31830 USA

**Keywords:** Blood pigments, Respiratory proteins, Transcriptome, Annelida

## Abstract

**Background:**

Despite extensive study on hemoglobins and hemocyanins, little is known about hemerythrin (Hr) evolutionary history. Four subgroups of Hrs have been documented, including: circulating Hr (cHr), myohemerythrin (myoHr), ovohemerythrin (ovoHr), and neurohemerythrin (nHr). Annelids have the greatest diversity of oxygen carrying proteins among animals and are the only phylum in which all Hr subgroups have been documented. To examine Hr diversity in annelids and to further understand evolution of Hrs, we employed approaches to survey annelid transcriptomes *in silico*.

**Results:**

Sequences of 214 putative Hr genes were identified from 44 annelid species in 40 different families and Bayesian inference revealed two major clades with strong statistical support. Notably, the topology of the Hr gene tree did not mirror the phylogeny of Annelida as presently understood, and we found evidence of extensive Hr gene duplication and loss in annelids. Gene tree topology supported monophyly of cHrs and a myoHr clade that included nHrs sequences, indicating these designations are functional rather than evolutionary.

**Conclusions:**

The presence of several cHrs in early branching taxa suggests that a variety of Hrs were present in the common ancestor of extant annelids. Although our analysis was limited to expressed-coding regions, our findings demonstrate a greater diversity of Hrs among annelids than previously reported.

**Electronic supplementary material:**

The online version of this article (doi:10.1186/s12862-017-0933-z) contains supplementary material, which is available to authorized users.

## Background

Metabolism in metazoans requires oxidation of organic molecules. Thus natural selection has presumably favored proteins that can reversibly bind and transport oxygen [1]. Such oxygen-binding proteins likely originated from enzymes whose primary function was to protect the body from oxygen toxicity, and, secondarily, these enzymes acquired the ability to carry oxygen molecules [2]. Several different classes of oxygen-carrying proteins, or respiratory pigments, are found across animal life. Although these molecules can reversibly bind oxygen, their binding affinities and evolutionary origins differ. In animals, oxygen-binding proteins are usually divided into two main groups: proteins that use iron to bind oxygen, including hemoglobins and hemerythrins, and hemocyanins that use copper [3]. Although hemoglobins and hemocyanins have been extensively investigated [4–8], knowledge on the evolutionary history of hemerythrins is limited [9]. Interestingly, medical sciences have increasingly been taking advantage of oxygen-binding proteins as blood substitutes [10, 11] or as carrier proteins for synthetic vaccines, (e.g., cancer vaccines; [12, 13]) making further study of oxygen binding protein diversity and evolution appealing.

Hemerythrins (Hrs) are a non-heme oligomeric protein family within the ‘four-helical up-and-down bundle’ fold and ‘all alpha proteins’ class according to the Structural Classification of Proteins database (SCOP) [14]. Oxygen-binding Hr proteins contain approximately 120 amino acid residues in a single domain and transport oxygen with the aid of two iron ions that bind directly to the polypeptide chain. Residues involved in iron binding include histidines (His) in positions 26 56, 75, 79, and 108, glutamic acid residue (Glu) in position 60 and aspartic acid residue (Asp) in position 113 (position numbers from *Themiste zostericola* in [15]). Presence of these signature residues indicates putative respiratory function for Hrs [16]. Functional Hr subunits usually form a homooctamer, although dimeric, trimeric, or tetrameric Hrs have been observed in some sipunculid species, including *Phascolosoma arcuatum*, *P. agasizii,* and *Siphonosoma funafuti* [17–19]. The crystal structure of Hrs consists of a bundle of four antiparallel α-helices (A, B, C, and D) formed by polypeptides: an A α-helix formed by 19 amino acid residues from position 19 to 38, B α-helix with 23 amino acids residues from position 43 to 65, C α-helix formed by 16 amino acids residues from position 72 to 88, and D α-helix formed by 20 amino acids residues from position 98 to 118, using *T. zostericola* as the reference sequence [9]. The core of active sites contains two iron atoms bridged by two carboxylate groups from aspartate and glutamate residues and an oxygen-containing ligand [20, 21]. Binding of oxygen apparently requires other currently unknown cellular factors since purified Hr, by itself, usually does not bind oxygen [22, 23]. Observed oxygen binding capacity is about 25% greater in Hrs than heme-based proteins, including hemoglobins [15].

Although Hr-like proteins have also been reported in prokaryotes [24–26] oxygen binding Hrs have only been reported from marine invertebrates belonging to Annelida (which include sipunculids; [27]) Brachiopoda, Priapulida, Bryozoa, and a single species of both Cnidaria (*Nematostella vectensis*) and Arthropoda (*Calanus finmarchicus*) [9, 18, 28, 29]. Given this phylogenetic breadth of animals, whether all metazoan Hrs share a common origin is debated [28, 29]. Overall, Hr proteins exhibit variation in their quaternary structure, and four groups have been reported based mainly on their primary structure and location within animal bodies [9]. Specifically, hemerythrins found in vascular tissue, referred to here as circulating hemerythrins (cHrs), and muscle-specific myohemerythrins (myoHr), have been better characterized compared to the other two hemerythrin groups, ovohemerythrins (ovoHr) and neurohemerythrins (nHr) [28, 30]. cHrs are polymeric intracellular proteins that occur inside nucleated cells, hemerythrocytes or pink blood cells located in coelomic fluid or vascular systems of Hr-bearing organisms [15]. In contrast, myoHrs are monomeric cytoplasmic proteins present in muscle cells of annelids [31]. The main difference between these groups is the presence of a five-codon insertion found in myoHr immediately before the D α-helix. Expression of myoHrs seems not to be restricted to cHrs-bearing organisms, considering that some annelid species possess both myoHrs and hemoglobins [32]. The other two groups of Hrs, ovohemerythrin (ovoHr) and neurohemerythrin (nHr), are also intracellular and non-circulating. ovoHr was identified in oocytes of the leech *Theromyzon tessulatum* and its presence during oogenesis possibly suggests a complex function in iron storage and detoxification [32, 33]. On the other hand, nHr was recently discovered in neural and non-neuronal tissues from the body wall of the leech *Hirudo medicinalis*, and it exhibits upregulation in response to septic injury [34]. Nevertheless, Vanin et al. suggested that nHr of leech may in fact be a myoHr [9]. Such diversification in Hr function may have involved gene duplications resulting in new proteins via neo- or subfunctionalization [32]. Moreover, ovoHrs and nHrs have only been reported in the literature a few times, and more studies are required to understand their function and evolution.

Annelids have the greatest diversity of oxygen-binding proteins among metazoans [35] and it is the only phylum from which all subtypes of Hr proteins have been documented [28]. While Hrs of annelids have been studied since the middle of the 20th century, until the 1990s, Hrs were recorded only from sipunculids and from a single polychaete family, Magelonidae [18, 36]. Later, Vanin et al. [9] found Hrs in a nereid and a leech and Bailly et al. [28] discovered Hrs genes in seven annelid species, suggesting Hrs are broadly distributed in annelids. Given the diversity of lifestyles among annelids known to have Hrs [29, 37], and the lack of information about Hrs in general [9], the occurrence and diversity of these molecules may be higher than currently recognized. Thus, to examine a wide diversity of annelid taxa for Hrs and to further understand how different forms of Hrs are evolutionarily related to each other, we employed approaches to survey Hrs from a diverse array of annelid transcriptomes *in silico*. We identified Hrs in 44 taxa and further describe the molecular diversity and evolution of Hrs in the light of annelid phylogeny [27, 38, 39]. Along with this, we assess whether described Hr subtypes consist of evolutionary lineages or result of independent adaptations to different organismal tissues.

## Results

Our bioinformatic analyses (Additional file 1) recovered a total of 415 unique nucleotide sequences of hemerythrin-like genes. Following translation Pfam domain evaluation and manual removal of sequences with less than 100 amino acid residues, 214 putative novel Hr genes were retained from all taxa examined in this study, representing 44 annelid species in 40 different families (Table 1). Novel Hr genes accession numbers for each species is available in Additional file 2. The number of expressed Hrs in a given species ranged from one in *Alciopa* sp*.*, *Cossura longocirrata*, *Enchytraeus albidus*, *Schizobranchia insignis*, and *Syllis* cf. *hyaline* to 11 in *Magelona berkeleyi* and *Phascolosoma agassizii*.

**Fig. 1:**
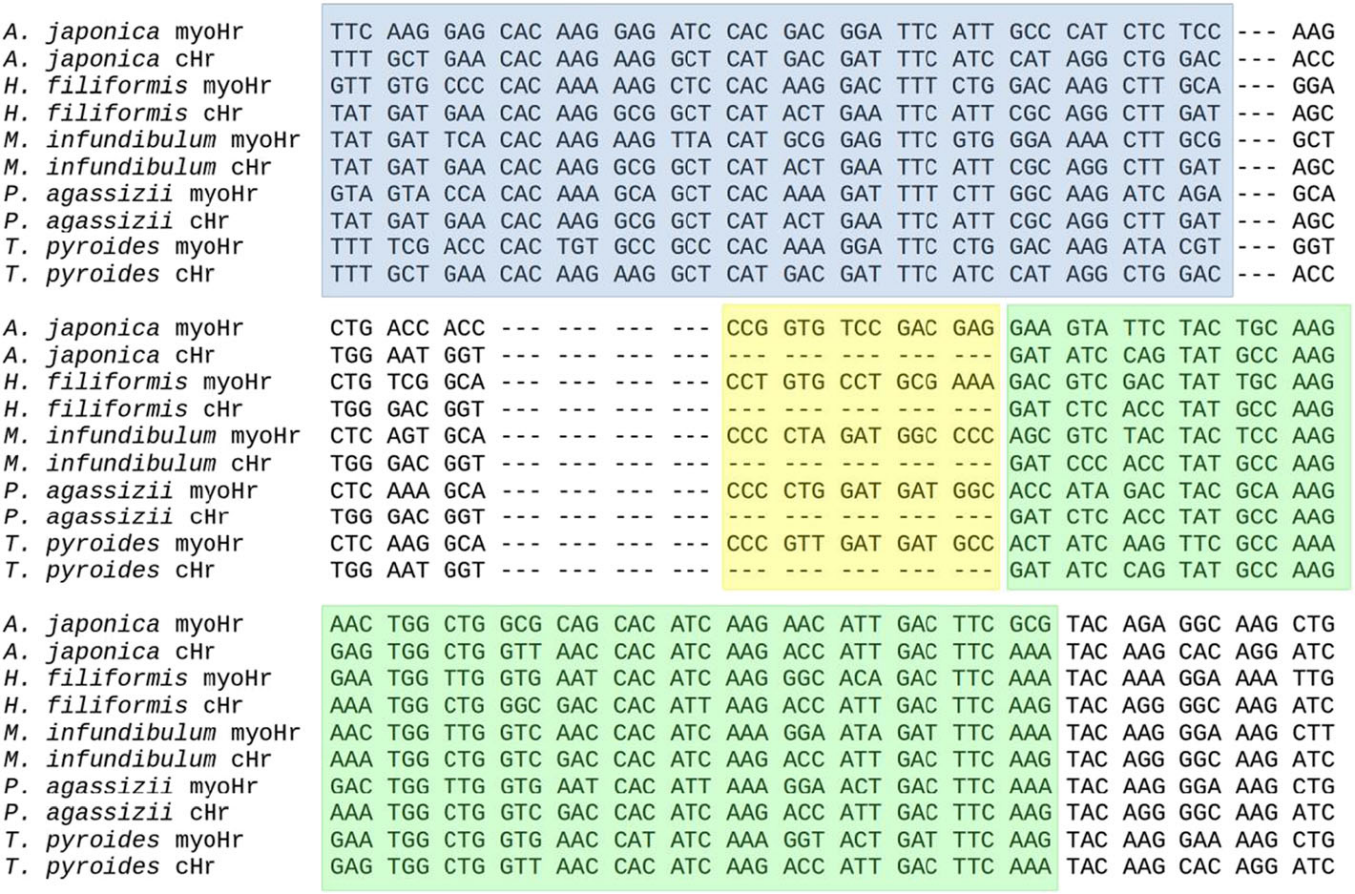
Alignment of nucleotide dataset of few species. Showing the five-codon insertion (yellow background) between C α-helix (blue background) and D α-helix (green background)

**Table 1:** List of taxa, including collection site, total number of base pairs sequenced, total number of contigs after assembly, number of putative Hr genes, and GenBank accession numbers. GenBank accession numbers for each Hr copy is indicated in Additional file 2

Species	Collection site	Total bp	Total contigs number	Hr genes number	Accession number
ALCIOPIDAE					
*Alciopa* sp.	N 33° 07.’ W 076° 06.4’	157,869,560	233,051	1	KY007275
ALVINELLIDAE					
*Paralvinella palmiformis* Desbruyères & Laubier, 1986	N 47° 56.9’ W 129° 05.9’	59,602,987	85,363	1	KY007423
AMPHINOMIDAE					
*Paramphinome jeffreysii* (McIntosh, 1868)	N 63° 30.8’ E 10° 25.0’	104,449,511	165,337	8	KY007424 to KY007431
APHRODITIDAE					
*Aphrodita japonica* Marenzeller, 1879	N 48° 28.6’ W 122° 58.7’	84,662,357	120,025	9	KY007279 to KY007287
ASPIDOSIPHONIDAE					
*Aspidosiphon laevis* de Quatrefages, 1865	N 09° 22.6’ W 82° 18.1’	120,601,137	168,072	4	KY007297 to KY007300
CAPITELLIDAE					
*Heteromastus filiformis* (Claparede, 1864)	N 41° 41.5’ W 070° 37.6’	94,824,555	148,196	8	KY007364 to KY007371
CHAETOPTERIDAE					
*Chaetopterus variopedatus* (Renier, 1804)	N 41° 41.5’ W 070° 43.5’	166,610,386	147,132	2	KY007307 and KY007309
*Mesochaetopterus taylori* Potts, 1914	N 48° 29.0’ W 123° 04.3’	86,521,966	83,209	3	KY007383 to KY007386
CHRYSOPETALIDAE					
*Arichlidon gathofi* Watson Russell, 2000	N 33° 59.6’ W 76° 42.1’	105,115,966	140,980	8	KY007288 to KY007296
CIRRATULIDAE					
*Chaetozone* sp.	N 66° 33.2’ E 33° 06.7’	85,730,053	143,587	4	KY007310 to KY007313
COSSURIDAE					
*Cossura longocirrata* Webster & Benedict, 1887	N 33° 29.4’ W 074° 48.0’	45,732,505	75,079	1	KY007319
ENCHYTRAEIDAE					
*Enchytraeus albidus* Henle, 1837	N 59^o^ 5.2' E 16^o^ 03.3'	13,345,974	22,776	1	KY007331
EUNICIDAE					
*Eunice pennata* (Müller, 1776)	N 39° 47.2’ W 70° 46.3’	59,429,144	93,814	5	KY007332 to KY007336
EUPHROSINIDAE					
*Euphrosine capensis* Kinberg, 1857	S 34° 09.9’ E 18° 26.0’	27,221,777	72,220	3	KY007337 to KY007339
FLABELLIGERIDAE					
*Poeobius meseres* Heath, 1930	N 36° 41.2’ W 122° 02.0’	25,964,726	70,078	2	KY007441 and KY007442
GLYCERIDAE					
*Glycera dibranchiata* Ehlers, 1868	N 41° 54.1’ W 070° 00.4’	51,282,233	101,455	3	KY007350 to KY007352
GOLFINGIIDAE					
*Thysanocardia nigra* (Ikeda, 1904)	N 48° 28.6’ W 122° 58.7’	57,399,340	58,011	6	KY007480 to KY007485
GONIADIDAE					
*Glycinde armigera* Moore, 1911	N 36° 23.0’ W 121° 57.9’	32,178,692	79,528	4	KY007353 to KY007356
HAPLOTAXIDAE					
*Delaya leruthi* (Hrabe, 1958)	N 43° 0.8’ E 01° 2.5’	93,863,431	118,020	7	KY007320 to KY007326
HESIONIDAE					
*Oxydromus pugettensis* (Johnson, 1901)	N 48° 34.3’ W 123° 10.1’	45,242,396	92,341	2	KY007421 and KY007422
LUMBRINERIDAE					
*Ninoe* sp.	N 35° 29.4’ W 074° 48.0’	120,256,564	151,183	5	KY007409 to KY007413
MAGELONIDAE					
*Magelona berkeleyi* Jones, 1971	N 36° 22.8’ W 121° 58.1’	16,339,407	50,123	10	KY007372 to KY007382
MALDANIDAE					
*Clymenella torquata* (Leidy, 1855)	N 41° 42.7’ W 070° 19.7’	62,661,529	111,567	5	KY007314 to KY007314
*Nicomache venticola* Blake & Hilbig, 1990	N 47° 57.0’ W 129° 05.9’	64,130,139	124,708	4	KY007405 to KY007408
NEPHTYIDAE					
*Nephtys incisa* Malmgren, 1865	N 40° 53.0’ W 070° 25.0’	126,720,409	188,338	6	KY007396 to KY007401
NEREIDIDAE					
*Alitta succinea* (Leuckart, 1847)	N 41° 54.0’ W 070° 00.3’	105,821,565	153,011	3	KY007402 to KY007404
OENONIDAE					
*Drilonereis* sp.	N 39° 54.1’ W 070° 35.1’	3,490,940	12,598	4	KY007327 to KY007330
*Oenone fulgida* (Savigny in Lamarck, 1818)	S 34° 37.0’ E 19° 21.4’	92973167	144,726	3	KY007414 to KY007416
ORBINIIDAE					
*Naineris laevigata* (Grube, 1855)	S 34° 35.0’ E 19° 20.9’	123,970,343	218,272	4	KY007392 to KY007395
OWENIIDAE					
*Galathowenia oculata* (Zachs, 1923)	N 66° 33.2’ E 33° 6.7’	128,195,375	179,612	10	KY007340 to KY007349
PECTINARIIDAE					
*Pectinaria gouldii* (Verrill, 1874)	N 41° 37.9’ W 070° 53.3’	63,132,019	92,091	9	KY007432 to KY007440
PHASCOLOSOMATIDAE					
*Phascolosoma agassizii* Keferstein, 1866	N 48° 31.2’ W 123° 01.0’	78,749,017	87,403	11	KY007443 to KY007453
PILARGIDAE					
*Ancistrosyllis groenlandica* McIntosh, 1879	N 40° 27.3’ W 070° 47.6’	39,327,753	94,924	3	KY007276 to KY007278
POLYNOIDAE					
*Halosydna brevisetosa* Kinberg, 1856	N 48° 29.5’ W 123° 01.1’	61,671,140	118,418	7	KY007357 to KY007363
RANDIELLIDAE					
*Randiella* sp.	S 14^o^ 40.0' E 145^o^ 27.1'	139,189,396	151,934	6	KY007454 to KY007459
SABELLIDAE					
*Myxicola infundibulum* (Montagu, 1808)	N 48° 28.6’ W 122° 58.7’	156,042,620	217,996	5	KY007387 to KY007391
*Schizobranchia insignis* Bush, 1905	N 48° 33.3’ W 122° 56.5’	55,085,979	102,002	1	KY007460
SPARGANOPHILIDAE					
*Sparganophilus* sp.	N 40° 50.3’ W 92° 5.3’	117,343,038	123,905	8	KY007461 to KY007468
SPIONIDAE					
*Boccardia proboscidea* Hartman, 1940	N 48° 29.2’ W 123° 04.1’	78,374,988	117,570	7	KY007301 to KY007307
STERNASPIDAE					
*Sternaspis scutata* Ranzani, 1817	N 48° 29.1’ W 123° 04.3’	81,147,455	115,096	3	KY007469 to KY007471
SYLLIDAE					
*Syllis* cf. *hyalina* Grube, 1863	S 34° 37.0’ E 19° 21.4’	76,801,405	106,283	1	KY007472
THEMISTIDAE					
*Themiste pyroides* (Chamberlin, 1919)	N 48° 21.4’ W 123° 43.4’	75,495,745	88,157	7	KY007473 to KY007479
TOMOPTERIDAE					
*Tomopteris* sp.	N 36° 41.2’ W 122° 02.0’	30,525,410	66,655	3	KY007486 to KY007488
Oligochaeta gen. sp. (unidentified Crassiclitellata - Place Kabary 2)	S 12° 59.0’ E 49° 17.4’	107,638,847	146,018	4	KY007417 to KY007420

Following trimming alignment of translated transcripts possessed 132 residue positions, with nearly all sequences, the exception being *Aphrodita japonica*, starting with a methionine residue. We decided to keep the apparently incomplete sequence from *A. japonica* due to its high similarity with the remaining sequences. All sequences in the alignment contained signature residues involved in iron binding, indicating putative respiratory function for these putative Hrs [16]. For the 214 sequences, 100 were unique and 114 identical for at least two species at the amino acid level.

Sequences were assembled into a final dataset containing 225 sequences being 214 new, two Hr sequences from *Lingula reevii*, a brachiopod, and nine annelid sequences previous used as “queries”, with 396 aligned nucleotides positions. Of these, 209 sequences contained a five-codon insertion between the C and D α-helices described for myoHr, but not cHrs (Fig. 1) [9]. Datasets supporting conclusions of this article are available in the Figshare repository, under DOIs: https://doi.org/10.6084/m9.figshare.3505883.v1 and https://doi.org/10.6084/m9.figshare.3505886.v1.

**Fig. 2:**
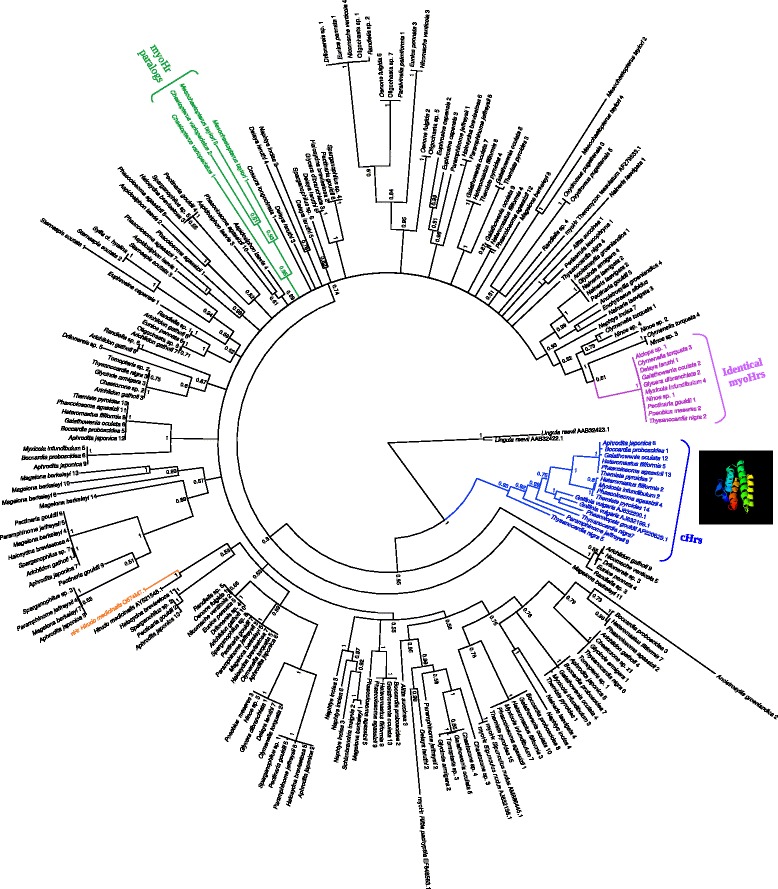
Bayesian tree using MrBayes 3.2.1 [51] rooted with two Hr sequences from *Lingula reevii*, a brachiopod. The blue clade represents the cHrs othologs sequences with a prediction of the consensus cHr sequence quaternary structure using I-TASSER 4.4 [61]. Black, purple, and green sequences represent the myoHr sequences and orange sequence is the only nHr from *Hirudo medicinalis*. The green clade shows the paralogs sequences from *C. variopedatus* and *M. taylori* and the purple clade represents the identical orthologs myoHr sequences. The number after the name of each sequence indicates the GenBank accession numbers for each Hr gene and it is indicated in Additional file 2: Table S1

Every species analyzed possessed at least one copy of myoHr gene. Bayesian inference rooted using two brachiopod Hr sequences revealed two major clades with one corresponding to cHrs clade (p.p. = 0.93; Fig. 2 blue clade) and the other supported monophyly for a myoHr clade (p.p. = 0.95; Fig. 2 black clade). The leech nHr sequence included in the dataset was found inside the myoHr clade (Fig. 2; orange). Within the myoHr clade low nodal support values and polytomies were found. However, such results are not uncommon for single protein or protein family trees [40].

Our analysis found 13 putative cHr sequences lacking the characteristic five-codon insertion before the D α-helix that define myoHrs, distributed across nine different families. Besides well-known records of cHrs in sipunculids, such as *Themiste pyroides* and *Phascolosoma agassizii*, we discovered cHrs in the sipunculid *Thysanocardia nigra* as well as six annelid families; Amphinomidae Aproditidae, Capitellidae, Oweniidae, Sabellidae, and Spionidae (Fig. 2; blue clade).

The topology of the Hr gene tree did not mirror recent phylogenies of Annelida based on phylogenomic datasets [27, 38, 39]. For example, we found 10 Hr sequences identical at the nucleotide level (Fig. 2, purple clade) belonging to distant annelid families indicating a strong conservation among those orthologs. Several of these sequences were prepared and sequenced at different times, making cross contamination unlikely. Those 10 identical sequences differed 28.54% (nucleotide level) from the consensus of all others myoHr sequences and the majority of nonsynonymous substitutions are concentrated in A and B α-helices.

Regarding paralogs multiple copies of Hr genes were found for several species, including two paralogs from both *Chaetopterus variopedatus* and *Mesochaetopterus taylori*, with these paralogs forming a monophyletic clade (p.p. = 0.85; Fig. 2; green clade). Both species were from Chaetopteridae suggesting a recent paralogous duplication [26]. Given the presence of multiple Hrs, apparently early annelids already contained several copies of Hr genes, with some paralogs arising later (as in *C. variopedatus* and *M. taylori*).

**Fig. 3:**
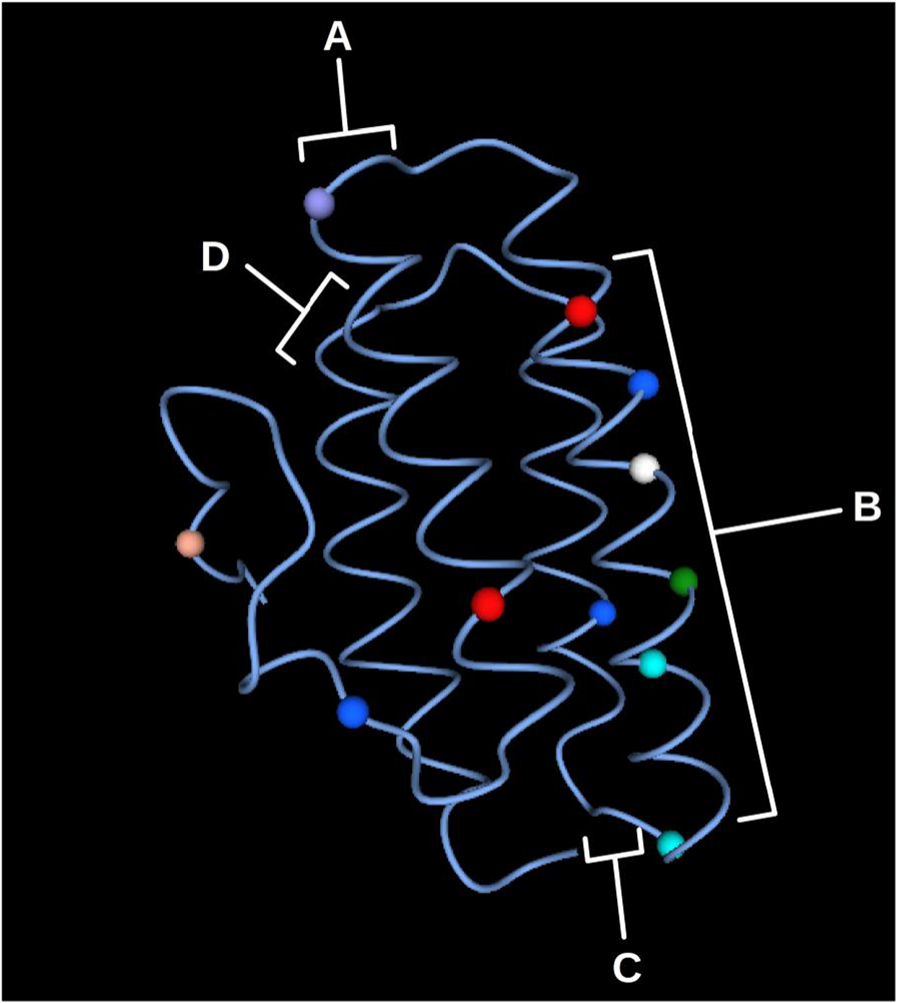
Hr showing differences in evolutionary rates between cHrs and myoHrs sequences calculated with DIVERGE [41] using a 0.7 cutoff. A and C α-helices presented one site each above the cutoff value, D α-helix did not present any sites, and B α-helix presented five sites. *Colored dots* represent sites above cutoff value, and different colors are only to aid illustration

Differences in evolutionary rates between cHrs and myoHrs sequences was accessed and relative rates of change in different positions were calculated using two different approaches. Sites with high variation were found not to have significantly different rates among inter-helices sites using DIVERGE [41]. For helix regions A and C α-helices had one site each with high evolutionary rate, while D α-helix did not have any sites with a high evolutionary rate, indicating a highly conservative region. At the same time, B α-helix had five sites, suggesting that this helix is evolving faster than others (Fig. 3). RELAX [42] was also used to assess differences in selection on cHrs relative to myoHrs while accounting for lineage-specific rate differences. Similar to the DIVERGE analyses, no significant differences (*P* = 0.218) were found between the two sets of genes. Thus, the gene duplication event leading to cHrs versus myoHrs does not seem to have been accompanied by a significant change in substitution rate or selection differences on the two gene lineages. Alternatively, any evidence of such a rate or selection difference could have been lost during the 500+ MY since these genes diverged.

## Discussion

As demonstrated here expression of Hr genes among annelids is much more common than previously reported, revealing unrecognized diversity of these genes in this phylum. All 44 of the examined species possessed actively transcribed myoHrs, while cHrs were less frequently recovered from these transcriptomes. Although this diversity and wide distribution of Hrs in annelids could, in part, be explained by the need to carry oxygen (Hr have approximately 25% greater oxygen affinity than hemoglobins; [15]) secondary functional specializations could also be important for driving diversification. For example, Hrs participate in iron storage, metal detoxification, and immunity in some annelids (e.g., *Theromyzon tessulatum*, *Hirudo medicinalis* and *Neanthes diversicolor*) [33, 34, 43]. Our findings build on recent publications demonstrating that sequence diversity among Hr-bearing species was larger than traditionally suspected [9, 28, 29]. However, those studies were based predominately on genomic data. In this case, use of transcriptomes shows that Hrs genes were present and expressed.

Bayesian phylogenetic reconstruction recovered monophyletic clades for annelid cHrs sequences as well as myoHrs sequences with strong support corroborating Vanin et al.’s [9] previous findings. The presence of multiple copies of myoHr genes across the annelid phylogeny implies these proteins have undergone several instances of gene duplication during their evolution, as previously reported for other bacterial, archeal, and eukaryotic taxa [26]. Moreover, the unexpected diversity of myoHrs could be associated with functional diversification of this gene, as observed for myoglobins [44] and also for Hrs involved in heavy metal detoxification and aspects of innate immunity [45].

Classification of specific Hr subtypes [28, 32–34] was not validated by the gene genealogy. Although our analyses used whole organisms (including reproductive and nerve tissues), our results failed to recover Hr proteins that corresponded to ovoHrs or nHrs. These categories, however, were described based on limited differences in amino acid sequence and do not reflect distinct monophyletic subgroups within the Hr gene family. Given this, recognizing only two primary types of Hrs, circulating Hrs (cHr) and non-circulating Hrs (ncHrs), is perhaps more appropriate. Although tentative, this reinterpretation deserves further consideration.

**Fig. 4:**
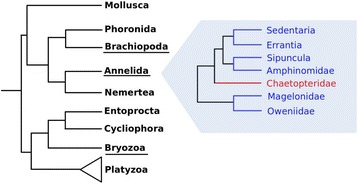
Lophotrochozoa and annelid relationships based on current knowledge [39, 62]. Underlined phyla represent the Hr-bearing representants. Annelid taxa in blue possess both cHr and myoHr genes and taxa in red possess just myoHr genes

Incongruence between our gene genealogy relative to current knowledge of annelid evolutionary history indicates that Hrs have a complex history which possibly involved events of gene losses and duplications, paralogs replacements and lateral gene transfer [26]. Although previous work supported the idea of a monomeric protein as an ancestral myoHr within annelids [9, 15], the presence of cHrs in *Paramphinome jeffreysii* (Amphinomidae), *Galathowenia oculata* (Oweniidae), *Phascolosoma agassizii*, *Themiste pyroides*, and *Thysanocardia nigra* (the last three belonging to Sipuncula), all members of lineages near the base of the annelid tree [27, 38, 39] (Fig. 4), indicates that both cHrs and myoHrs were likely present in the ancestor of annelids, which date back to the Cambrian [46]. Interestingly, Hrs from multiple species, representing hundreds of millions of years of evolution, possessed identical amino acid, and in some cases nucleotide, sequences (Fig. 2, purple clade), suggesting a level of conservation and selection orders of magnitude greater than most of the genome. Additional studies across metazoan together with studies of the gene structure of Hr proteins and physiological aspects of organisms are the next important steps toward a better understanding of the evolutionary patterns involved in this family of oxygen carrying proteins.

## Conclusions

Our findings demonstrate that sequence diversity among Hr-bearing annelid species is much greater than traditionally suspected and that many of these Hrs are actively expressed. There are two primary types of Hrs circulating Hrs (cHr) and non-circulating Hrs (ncHrs), instead of the four subtypes reported in the literature. Incongruence between our gene genealogy relative to current knowledge of annelid evolutionary history indicates that Hrs have a complex history. Our findings indicate that both cHrs and myoHrs were likely present in the ancestor of annelids, as both subtypes occur in all lineages near the base of the annelid tree.

## Methods

### Sample collection

Information on species employed herein is provided in Table 1. Transcriptomes of these species were collected as part of the WormNet II project to resolve annelid phylogeny and were collected with a variety of techniques, including intertidal sampling, dredge and box cores. Upon collection, all samples were either preserved in RNALater or frozen at −80 °C.

### Data collection & sequence assembly

RNA extraction, cDNA preparation and high-throughput sequencing generally followed Kocot et al. [47] and Whelan et al. [48]. Briefly, total RNA was extracted from either whole animals (for small specimens) or the body wall and coelomic region (for larger specimens). After extraction, RNAs were purified using TRIzol (Invitrogen) or the RNeasy kit (Qiagen) with on-column DNase digestion, respectively. The SMART cDNA Library Construction Kit (Clonetech) was utilized to reverse transcribe single stranded RNA template. Double stranded cDNA synthesis was completed with The Advantage 2 PCR system (Clontech). Libraries were barcoded and sequenced with Illumina technology by The Genomic Services Lab at the Hudson Alpha Institute (Huntsville, Alabama, USA). Because transcriptomic sequencing was conducted from 2012–2015, Paired End (PE) runs were of 100 bp or 125 bp lengths, utilizing either v3 or v4 chemistry on Illumina HiSeq 2000 or 2500 platforms (San Diego, California). To facilitate sequence assembly, paired-end transcriptome data were digitally normalized to an average k-mer coverage of 30 using normalize-by-median.py [49] and assembled using Trinity r2013-02-25 with default settings [50].

### Data mining and gene identification

Two approaches were utilized in searching transcriptomic data for putative Hr genes *in silico*. The first approach employed BLASTX [51] at an e-value cutoff of 10^−6^ to compare each assembled transcriptome contig (‘queries’) to a protein database composed of 21 protein sequence from the National Center for Biotechnology (NCBI) database (Additional file 3: Table S2) of at least 110 amino acid residues and previously identified as annelid Hrs (*n* = 11), myoHrs (*n* = 9), or nHr (*n* = 1). Sequences of ovoHrs were not included since there were only two relatively short (18 amino acid residues each) sequences available from NCBI. The BLASTX approach, rather than a tBLASTn, assured that any transcriptome contig with a significant ‘hit’ to an Hr would be further evaluated in the pipeline. Contigs recovered from initial BLAST searches were then utilized in BLASTX searches against the NCBI protein database (minimum e-value of 10^−10^) and only top hits longer than 300 nucleotides retained. These were considered putative cHr, myoHr or nHr genes, as appropriate.

The second approach processed the transcriptomic data through the Trinotate annotation pipeline (http://trinotate.github.io/) [50], which utilizes a BLAST-based approach to provide, among others, GO (The Gene Ontology Consortium) annotation [52]. Recovered sequences were verified by the functional annotation they received. Transcripts annotated as hemerythrins, using the 10^−6^ e-value cutoff obtained by using BLASTX, were also considered putative hemerythrin-like gene orthologs.

Contigs putatively identified as Hr genes by the two approaches were subsequently translated into amino acids using TransDecoder with default settings [53]. Since TransDecoder can produce multiple open reading frames (ORFs), all translations were additionally subject to a Pfam Domain evaluation using the EMBL-EBI database with an e-value cutoff of 10^−5^. Translations returning an Hr-like Pfam domain and that were longer than 100 amino acids residues were retained for subsequent analyses. Moreover, we manually evaluated the presence of residues involved in iron binding, which are: histidine residues (His) in positions 26, 56, 75, 79, and 108; glutamic acid residue (Glu) in position 60; and aspartic acid residue (Asp) in position 113, numbered by reference sequence *T. zostericola*. Presence of these signature residues indicates putative respiratory function for Hrs. Transcripts passing the criteria described above were considered Hr genes (Table 1). ovoHr has been just reported once for a single species and there is no complete sequence available at GenBank, so we are not able to investigate if ovoHr is present in our dataset.

### Sequence alignment

The protein dataset consisted of 225 sequences, including two Hr sequences from *Lingula reevii*, a brachiopod, nine annelid sequences previous used as “queries” (Additional file 3: Table S2), and a remaining 214 sequences from translated transcripts (Additional file 4). All sequences were initially aligned with MAFFT using the “accurate E-INS-i” algorithm [54], followed by visual inspection and manual curation in order to remove spuriously aligned sequences based on similarity to the protein alignment as a whole. Subsequently, ends of aligned sequences were manually trimmed in Geneious 8.1.6 [55] to exclude 5’residues leading to the putative start codon and 3’ residues following the first seven amino acids subsequent to the end of the D α-helix.

In order to employ nucleotide sequences in the phylogenetic analysis, an alignment from corresponding aligned protein sequences (Additional file 4) was performed using PAL2NAL [56]. The resulting nucleotide alignment was used for all subsequent analyses (Additional file 5).

### Phylogenetic analysis

JModelTest2 was applied to carry out statistical selection of best-fit models of nucleotide substitution for the dataset using the Akaike and Bayesian Information Criteria (AIC and BIC, respectively) methods [57]. Bayesian phylogenetic inference was performed with MrBayes 3.2.1 [58] using the GTR + G substitution model. Two independent runs with four Metropolis-coupled chains were run for 10^7^ generations, sampling the posterior distribution every 500 generations. In order to confirm if chains achieved stationary and determine an appropriate burn-in, we evaluated trace plots of all MrBayes parameter output in Tracer v1.6 [59]. The first 25% of samples were discarded as burn-in and a majority rule consensus tree generated using MrBayes. Bayesian posterior probabilities were used for assessing statistical support of each bipartition.

Two alternative approaches were used to root the Hr gene genealogy. Firstly, the tree inferred in MrBayes was rooted using two Hr sequences from *Lingula reevii* (AAB32422.1 and AAB32423.1) as outgroup. We also inferred the root of the tree using using BEAST 1.8.3 [60] to infer a rooted tree of Hrs under the strict molecular clock. This was done because we were unable to decisively rule out the possibility that Hr sequences from *Lingula reevii* were closely evolutionarily related to one of the annelid Hr lineages. The strategy using BEAST is similar to midpoint rooting, although the Bayesian implementation in BEAST allows for a more flexible treatment of the evolutionary rate via a normal prior with mean and standard deviation equal to 1. Moreover, tree topology was jointly estimated. In BEAST, we adopted the same substitution model settings used in MrBayes and the MCMC algorithm was run for 50,000,000 generations and sampled every 1,000th generation, with 50% of the run discarded as burn-in. Trees was summarized in TreeAnnotator 1.8.3 [60] and Markov chain stationarity was assessed in Tracer by ESS values > 1,000. Since the inferred root node separated *Lingula reevii* sequences from annelid sequences with 0.86 posterior probability, the results are reported using the gene genealogy rooted with outgroup.

### Evolutionary rate analyses

The protein alignment (Additional file 4) was used in DIVERGE [41] to examine site-specific shifted evolutionary rates and assesses whether there has been a significant change in evolution rate after duplication or speciation events by calculating the coefficient of divergence (θD) and determining if the null hypothesis of no functional divergence between myoHrs and cHrs could be statistically rejected. We employed a cutoff of 0.7 for detection of site-specific shifted evolutionary rates.

Additionally, we used RELAX [42] to detect changes in selection intensity between cHRs and myoHrs based on aligned nucleotide data (Additional file 5). Rather than just looking in *dN/dS* ratios averaged across branches using a ‘branch-site’ model approach, RELAX examines *dN/dS* ratios along each branch at a given site by drawing values from a discrete distribution of *dN/dS* ratios independent of other branches. In addition to assessing values in a branch independent manner, RELAX results are analyzed within a phylogenetic framework.

## Additional files

Additional file 1: Flow chart of bioinformatics pipeline. Rounded purple rectangles represent input/output files, orange ovals represent software or scripts, and the green hexagon represents a step which involving manual evaluation. Nine annelid Hrs sequences previous used as query and two *Lingula* (Brachipoda) sequences from Genbank (Additional file 3) were also included in the dataset. (DOC 1870 kb)

Additional file 2: Hr genes with their respective accession numbers. Novel Hr genes accession numbers for each species. From the manuscript of Costa-Paiva et al. BMC Evolutionary Biology. (DOC 217 kb)

Additional file 3: Outgroup and query sequences used to search assembled translated transcriptomes. Query seqeunces used to search transcriptomes. The Hr sequence for H. medicinalis possesses the myoHr five codon insertion between the C and D α-helix, so in this work we considered it a myoHr. Sequences in bold was also included in the dataset previous to the alignment. From the manuscript of Costa-Paiva et al. BMC Evolutionary Biology (DOC 53 kb)

Additional file 4: The amino acid alignment used in analyses. The nucleotide alignment used for all subsequent analyses. (TXT 31 kb)

Additional file 5: The nucleotide alignment used for analyses. (TXT 89 kb)

## Abbreviations

cHr: Circulating hemerythrin
Hr: Hemerythrin
myoHr: Myohemerythrin
ncHr: Non-circulating hemerythrin
nHr: Neurohemerythrin
ovoHr: Ovohemerythrin
