# Supplementary material for: Discovery and evolution of novel hemerythrin genes in annelid worms
Source: BMC Evol Biol. 2017 Mar 23;17:85. doi: 10.1186/s12862-017-0933-z (PMC5363010; doi:10.1186/s12862-017-0933-z)
Supplement: Supplementary file 1 — Flow chart of bioinformatics pipeline. Rounded purple rectangles represent input/output files, orange ovals represent software or scripts, and the green hexagon represents a step which involving manual evaluation. Nine annelid Hrs sequences previous used as query and two Lingula (Brachipoda) sequences from Genbank (Additional file 3) were also included in the dataset. (DOC 1870 kb) [file 12862_2017_933_MOESM1_ESM.doc]

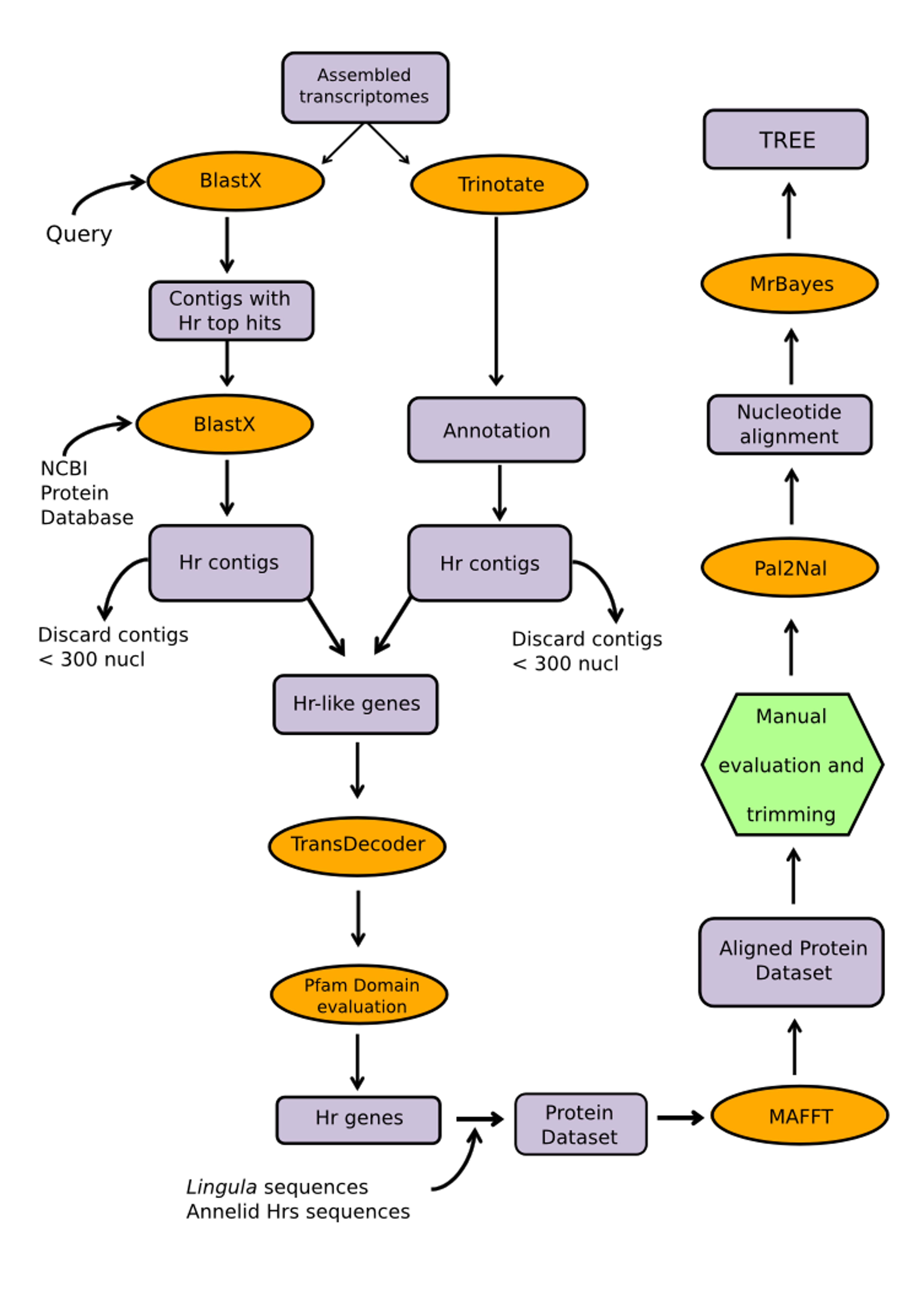


**Additional File 1 - Flow chart of bioinformatics pipeline. Rounded purple rectangles represent input/output files, orange ovals represent software or scripts, and the green hexagon represents a step which involving manual evaluation. Nine annelid Hrs sequences previous used as query and two *Lingula* (Brachipoda) sequences from Genbank (Additional File 3) were also included in the dataset. From the manuscript of Costa-Paiva et al. BMC Evolutionary Biology.**
