# Supplementary material for: Discovery and evolution of novel hemerythrin genes in annelid worms
Source: BMC Evol Biol. 2017 Mar 23;17:85. doi: 10.1186/s12862-017-0933-z (PMC5363010; doi:10.1186/s12862-017-0933-z)
Supplement: Supplementary file 2 — Hr genes with their respective accession numbers. Novel Hr genes accession numbers for each species. From the manuscript of Costa-Paiva et al. BMC Evolutionary Biology. (DOC 217 kb) [file 12862_2017_933_MOESM2_ESM.doc]

**Additional File 2. Novel Hr genes accession numbers for each species. From the manuscript of Costa-Paiva et al. BMC Evolutionary Biology.**

| **Species** |  |
| --- | --- |
| ALCIOPIDAE |  |
| *Alciopa* sp. | KY007275 |
| ALVINELLIDAE |  |
| *Paralvinella palmiformis* | KY007421 |
| AMPHINOMIDAE |  |
| *Paramphinome jeffreysii* 1 | KY007424 |
| *Paramphinome jeffreysii* 2 | KY007425 |
| *Paramphinome jeffreysii* 3 | KY007426 |
| *Paramphinome jeffreysii* 4 | KY007427 |
| *Paramphinome jeffreysii* 5 | KY007428 |
| *Paramphinome jeffreysii* 6 | KY007429 |
| *Paramphinome jeffreysii* 8 | KY007430 |
| *Paramphinome jeffreysii* 9 | KY007431 |
| APHRODITIDAE |  |
| *Aphrodita japonica* 2 | KY007279 |
| *Aphrodita japonica* 3 | KY007280 |
| *Aphrodita japonica* 5 | KY007281 |
| *Aphrodita japonica* 6 | KY007282 |
| *Aphrodita japonica* 7 | KY007283 |
| *Aphrodita japonica* 8 | KY007284 |
| *Aphrodita japonica* 9 | KY007285 |
| *Aphrodita japonica* 12 | KY007286 |
| *Aphrodita japonica* 13 | KY007287 |
| ASPIDOSIPHONIDAE |  |
| *Aspidosiphon laevis* 1 | KY007297 |
| *Aspidosiphon laevis* 2 | KY007298 |
| *Aspidosiphon laevis* 3 | KY007299 |
| *Aspidosiphon laevis* 4 | KY007300 |
| CAPITELLIDAE |  |
| *Heteromastus filiformis* 2 | KY007364 |
| *Heteromastus filiformis* 3 | KY007365 |
| *Heteromastus filiformis* 4 | KY007366 |
| *Heteromastus filiformis* 5 | KY007367 |
| *Heteromastus filiformis* 6 | KY007368 |
| *Heteromastus filiformis* 7 | KY007369 |
| *Heteromastus filiformis* 8 | KY007370 |
| *Heteromastus filiformis* 9 | KY007371 |
| CHAETOPTERIDAE |  |
| *Chaetopterus variopedatus* 1 | KY007308 |
| *Chaetopterus variopedatus* 2 | KY007309 |
| *Mesochaetopterus taylorii* 1 | KY007383 |
| *Mesochaetopterus taylorii* 2 | KY007384 |
| *Mesochaetopterus taylorii* 3 | KY007385 |
| *Mesochaetopterus taylorii* 4 | KY007386 |
| CHRYSOPETALIDAE |  |
| *Arichlidon gathofi* 1 | KY007288 |
| *Arichlidon gathofi* 3 | KY007289 |
| *Arichlidon gathofi* 4 | KY007290 |
| *Arichlidon gathofi* 5 | KY007291 |
| *Arichlidon gathofi* 6 | KY007292 |
| *Arichlidon gathofi* 7 | KY007293 |
| *Arichlidon gathofi* 8 | KY007294 |
| *Arichlidon gathofi* 9 | KY007295 |
| *Arichlidon gathofi* 10 | KY007296 |
| CIRRATULIDAE |  |
| *Chaetozone* sp. 1 | KY007310 |
| *Chaetozone* sp. 2 | KY007311 |
| *Chaetozone* sp. 3 | KY007312 |
| *Chaetozone* sp. 4 | KY007313 |
| COSSURIDAE |  |
| *Cossura longocirrata* | KY007319 |
| ENCHYTRAEIDAE |  |
| *Enchytraeus albidus* | KY007331 |
| EUNICIDAE |  |
| *Eunice pennata* 1 | KY007332 |
| *Eunice pennata* 3 | KY007333 |
| *Eunice pennata* 4 | KY007334 |
| *Eunice pennata* 5 | KY007335 |
| *Eunice pennata* 6 | KY007336 |
| EUPHROSINIDAE |  |
| *Euphrosine capensis* 1 | KY007337 |
| *Euphrosine capensis* 2 | KY007338 |
| *Euphrosine capensis* 3 | KY007339 |
| FLABELLIGERIDAE |  |
| *Poeobius meseres* 2 | KY007441 |
| *Poeobius meseres* 3 | KY007442 |
| GLYCERIDAE |  |
| *Glycera dibranchiata* 1 | KY007350 |
| *Glycera dibranchiata* 2 | KY007351 |
| *Glycera dibranchiata* 3 | KY007352 |
| GOLFINGIIDAE |  |
| *Thysanocardia nigra* 2 | KY007480 |
| *Thysanocardia nigra* 3 | KY007481 |
| *Thysanocardia nigra* 4 | KY007482 |
| *Thysanocardia nigra* 5 | KY007483 |
| *Thysanocardia nigra* 6 | KY007484 |
| *Thysanocardia nigra* 7 | KY007485 |
| GONIADIDAE |  |
| *Glycinde armigera* 1 | KY007353 |
| *Glycinde armigera* 2 | KY007354 |
| *Glycinde armigera* 3 | KY007355 |
| *Glycinde armigera* 4 | KY007356 |
| HAPLOTAXIDAE |  |
| *Delaya leruthi* 1 | KY007320 |
| *Delaya leruthi* 2 | KY007321 |
| *Delaya leruthi* 3 | KY007322 |
| *Delaya leruthi* 4 | KY007323 |
| *Delaya leruthi* 5 | KY007324 |
| *Delaya leruthi* 6 | KY007325 |
| *Delaya leruthi* 7 | KY007326 |
| HESIONIDAE |  |
| *Oxydromus pugettensis* 3 | KY007421 |
| *Oxydromus pugettensis* 5 | KY007422 |
| LUMBRINERIDAE |  |
| *Ninoe* sp. 1 | KY007409 |
| *Ninoe* sp. 2 | KY007410 |
| *Ninoe* sp. 3 | KY007411 |
| *Ninoe* sp. 4 | KY007412 |
| *Ninoe* sp. 5 | KY007413 |
| MAGELONIDAE |  |
| *Magelona berkeleyi* 1 | KY007372 |
| *Magelona berkeleyi* 4 | KY007373 |
| *Magelona berkeleyi* 5 | KY007374 |
| *Magelona berkeleyi* 6 | KY007375 |
| *Magelona berkeleyi* 7 | KY007376 |
| *Magelona berkeleyi* 8 | KY007377 |
| *Magelona berkeleyi* 9 | KY007378 |
| *Magelona berkeleyi* 10 | KY007379 |
| *Magelona berkeleyi* 11 | KY007380 |
| *Magelona berkeleyi* 13 | KY007381 |
| *Magelona berkeleyi* 14 | KY007382 |
| MALDANIDAE |  |
| *Clymenella torquata* 1 | KY007314 |
| *Clymenella torquata* 2 | KY007315 |
| *Clymenella torquata* 3 | KY007316 |
| *Clymenella torquata* 4 | KY007317 |
| *Clymenella torquata* 5 | KY007318 |
| *Nicomache venticola* 2 | KY007405 |
| *Nicomache venticola* 3 | KY007406 |
| *Nicomache venticola* 4 | KY007407 |
| *Nicomache venticola* 5 | KY007408 |
| NEPHTYIDAE |  |
| *Nephtys incisa* 2 | KY007396 |
| *Nephtys incisa* 3 | KY007397 |
| *Nephtys incisa* 4 | KY007398 |
| *Nephtys incisa* 5 | KY007399 |
| *Nephtys incisa* 6 | KY007400 |
| *Nephtys incisa* 7 | KY007401 |
| NEREIDIDAE |  |
| *Alitta succinea* 1 | KY007402 |
| *Alitta succinea* 2 | KY007403 |
| *Alitta succinea* 3 | KY007404 |
| OENONIDAE |  |
| *Drilonereis* sp. 1 | KY007327 |
| *Drilonereis* sp. 3 | KY007328 |
| *Drilonereis* sp. 4 | KY007329 |
| *Drilonereis* sp. 5 | KY007330 |
| *Oenone fulgida* 1 | KY007414 |
| *Oenone fulgida* 2 | KY007415 |
| *Oenone fulgida* 5 | KY007416 |
| ORBINIIDAE |  |
| *Naineris laevigata* 1 | KY007392 |
| *Naineris laevigata* 2 | KY007393 |
| *Naineris laevigata* 3 | KY007394 |
| *Naineris laevigata* 4 | KY007395 |
| OWENIIDAE |  |
| *Galathowenia oculata* 2 | KY007340 |
| *Galathowenia oculata* 4 | KY007341 |
| *Galathowenia oculata* 5 | KY007342 |
| *Galathowenia oculata* 6 | KY007343 |
| *Galathowenia oculata* 7 | KY007344 |
| *Galathowenia oculata* 8 | KY007345 |
| *Galathowenia oculata* 9 | KY007346 |
| *Galathowenia oculata* 10 | KY007347 |
| *Galathowenia oculata* 12 | KY007348 |
| *Galathowenia oculata* 13 | KY007349 |
| PECTINARIIDAE |  |
| *Pectinaria gouldii* 1 | KY007432 |
| *Pectinaria gouldii* 2 | KY007433 |
| *Pectinaria gouldii* 3 | KY007434 |
| *Pectinaria gouldii* 4 | KY007435 |
| *Pectinaria gouldii* 5 | KY007436 |
| *Pectinaria gouldii* 6 | KY007437 |
| *Pectinaria gouldii* 7 | KY007438 |
| *Pectinaria gouldii* 8 | KY007439 |
| *Pectinaria gouldii* 9 | KY007440 |
| PHASCOLOSOMATIDAE |  |
| *Phascolosoma agassizii* 1 | KY007443 |
| *Phascolosoma agassizii* 2 | KY007444 |
| *Phascolosoma agassizii* 4 | KY007445 |
| *Phascolosoma agassizii* 5 | KY007446 |
| *Phascolosoma agassizii* 7 | KY007447 |
| *Phascolosoma agassizii* 8 | KY007448 |
| *Phascolosoma agassizii* 9 | KY007449 |
| *Phascolosoma agassizii* 10 | KY007450 |
| *Phascolosoma agassizii* 11 | KY007451 |
| *Phascolosoma agassizii* 12 | KY007452 |
| *Phascolosoma agassizii* 13 | KY007453 |
| PILARGIDAE |  |
| *Ancistrosyllis groenlandica* 1 | KY007276 |
| *Ancistrosyllis groenlandica* 2 | KY007277 |
| *Ancistrosyllis groenlandica* 4 | KY007278 |
| POLYNOIDAE |  |
| *Halosydna brevisetosa* 1 | KY007357 |
| *Halosydna brevisetosa* 2 | KY007358 |
| *Halosydna brevisetosa* 3 | KY007359 |
| *Halosydna brevisetosa* 4 | KY007360 |
| *Halosydna brevisetosa* 5 | KY007361 |
| *Halosydna brevisetosa* 6 | KY007362 |
| *Halosydna brevisetosa* 7 | KY007363 |
| RANDIELLIDAE |  |
| *Randiella* sp. 1 | KY007454 |
| *Randiella* sp. 2 | KY007455 |
| *Randiella* sp. 3 | KY007456 |
| *Randiella* sp. 4 | KY007457 |
| *Randiella* sp. 5 | KY007458 |
| *Randiella* sp. 6 | KY007459 |
| SABELLIDAE |  |
| *Myxicola infundibulum* 2 | KY007387 |
| *Myxicola infundibulum* 3 | KY007388 |
| *Myxicola infundibulum* 4 | KY007389 |
| *Myxicola infundibulum* 5 | KY007390 |
| *Myxicola infundibulum* 7 | KY007391 |
| *Schizobranchia insignis* | KY007460 |
| SPARGANOPHILIDAE |  |
| *Sparganophilus* sp. 1 | KY007461 |
| *Sparganophilus* sp. 2 | KY007462 |
| *Sparganophilus* sp. 3 | KY007463 |
| *Sparganophilus* sp. 4 | KY007464 |
| *Sparganophilus* sp. 5 | KY007465 |
| *Sparganophilus* sp. 6 | KY007466 |
| *Sparganophilus* sp. 7 | KY007467 |
| *Sparganophilus* sp. 8 | KY007468 |
| SPIONIDAE |  |
| *Boccardia proboscidea* 1 | KY007301 |
| *Boccardia proboscidea* 2 | KY007302 |
| *Boccardia proboscidea* 3 | KY007303 |
| *Boccardia proboscidea* 5 | KY007304 |
| *Boccardia proboscidea* 6 | KY007305 |
| *Boccardia proboscidea* 7 | KY007306 |
| *Boccardia proboscidea* 8 | KY007307 |
| STERNASPIDAE |  |
| *Sternaspis scutata* 1 | KY007469 |
| *Sternaspis scutata* 2 | KY007470 |
| *Sternaspis scutata* 3 | KY007471 |
| SYLLIDAE |  |
| *Syllis* cf. *hyalina* | KY007472 |
| THEMISTIDAE |  |
| *Themiste pyroides* 1 | KY007473 |
| *Themiste pyroides* 3 | KY007474 |
| *Themiste pyroides* 4 | KY007475 |
| *Themiste pyroides* 7 | KY007476 |
| *Themiste pyroides* 13 | KY007477 |
| *Themiste pyroides* 14 | KY007478 |
| *Themiste pyroides* 15 | KY007479 |
| TOMOPTERIDAE |  |
| *Tomopteris* sp. 1 | KY007486 |
| *Tomopteris* sp. 2 | KY007487 |
| *Tomopteris* sp. 3 | KY007488 |
|  |  |
| Oligochaeta gen. sp. 1 | KY007417 |
| Oligochaeta gen. sp. 5 | KY007418 |
| Oligochaeta gen. sp. 7 | KY007419 |
| Oligochaeta gen. sp. 8 | KY007420 |
