# Supplementary material for: Discovery and evolution of novel hemerythrin genes in annelid worms
Source: BMC Evol Biol. 2017 Mar 23;17:85. doi: 10.1186/s12862-017-0933-z (PMC5363010; doi:10.1186/s12862-017-0933-z)
Supplement: Supplementary file 3 — Outgroup and query sequences used to search assembled translated transcriptomes. Query seqeunces used to search transcriptomes. The Hr sequence for H. medicinalis possesses the myoHr five codon insertion between the C and D α-helix, so in this work we considered it a myoHr. Sequences in bold was also included in the dataset previous to the alignment. From the manuscript of Costa-Paiva et al. BMC Evolutionary Biology (DOC 53 kb) [file 12862_2017_933_MOESM3_ESM.doc]

**Additional File 3: Query seqeunces used to search transcriptomes. The Hr sequence for *H. medicinalis* possesses the myoHr five codon insertion between the C and D α-helix, so in this work we considered it a myoHr. Sequences in bold was also included in the dataset previous to the alignment. From the manuscript of Costa-Paiva et al. BMC Evolutionary Biology.**

| Taxon | Protein | GenBank accession number  Protein | GenBank accession number  cds | Reference |
| --- | --- | --- | --- | --- |
| Annelida |  |  |  |  |
| ***Golfinia vulgaris***  **Golfingiidae** | **Hemerythrin subunit 2, partial**  **Hemerythrin subunit 1** | **CAG14946.1**  **CAG14945.1** | **AJ632200.1**  **AJ632199.1** | Vanin et al, 2006  Vanin et al, 2006 |
| *Hediste diversicolor*  Nereididae | Myohemerythrin | P22761.1 |  | Tagaki & Cox, 1991 |
| ***Hirudo medicinalis***  **Hirudinidae** | **Hemerythrin (myoHr)**  **Neurohemerythrin** | **AAS49167.1**  **Q674M7.1** | **AY521548.1** | Vergote et al, 2004  Vergote et al, 2004 |
| ***Periserrula leucophryna***  **Nereididae** | **Hemerythrin**  **(macronuclear)** | **AAQ63634.1** | **AY312845.1** | Choi et al, 2003 |
| ***Phascolopsis gouldii***  **Sipunculidae** | Myohemerythrin-1  Myohemerythrin  **Hemerytrin** | P27686.2  AAB22826.1  **AAF25482.1** | **AF220529.1** | Long et al, 1992  Long et al, 1992  Farmer et al, 2000 |
| ***Phascolosoma esculenta***  **Phascolosomatidae** | **Hemerythrin** | **ACA62947.1** | **EU368753.1** | Liu et al, 2013 |
| ***Riftia pachyptila***  **Siboglinidae** | **Myohemerythrin** | **ABW24415.1** | **EF648563.1** | Sanchez et al, 2007 |
| *Siphonosoma cumanense*  Sipunculidae | Hemerythrin | P22766.1 |  | Uchida et al, 1990 |
| ***Sipunculus nudus***  **Sipunculidae** | **Myohemerythrin**  **Myohemerythrin**  Hemerythrin  Hemerythrin  Hemerythrin | **CAP08292.1**  **CAG14944.1**  CAP08291.1  CAG14943.1  CAF74912.1 | **AM886445.1**  **AJ632198.1**  AM886444.1  AJ632197.1  AJ632021.1 | Bailly et al, 2008  Vanin et al, 2006  Bailly et al, 2008  Vanin et al, 2006  Vanin et al, 2006 |
| *Themiste dyscritum*  Themistidae | Hemerythrin | P02246.1 |  | Loehr et al, 1978 |
| *Themiste zostericola*  Themistidae | Myohemerythrin  Myohemerythrin | 1A7E_A  1A7D_A |  | Martins et al, 1997  Martins et al, 1997 |
| ***Theromyzon tessulatum***  **Glossiphoniidae** | **Myohemerythrin** | **AAG01808.1** | **AF279333.1** | Coutte et al, 2001 |
| Brachiopoda  (outgroup) |  |  |  |  |
| ***Lingula reevii***  **Lingulidae** | **Hemerythrin**  **Hemerythrin** | **AAB32423.1**  **AAB32422.1** |  | Negri et al, 1994  Negri et al, 1994 |
